# Potato and sweetpotato breeding at the International Potato Center: approaches, outcomes and the way forward

**DOI:** 10.1007/s00122-023-04515-7

**Published:** 2023-12-19

**Authors:** Hannele Lindqvist-Kreuze, Merideth Bonierbale, Wolfgang J. Grüneberg, Thiago Mendes, Bert De Boeck, Hugo Campos

**Affiliations:** 1 International Potato Center, Lima 12, 1558 Apartado, Peru; 2 Present Address: Calle Bolivia, 12 Manilva, 29690 Malaga, Spain

## Abstract

Root and tuber crop breeding is at the front and center of CIP’s science program, which seeks to develop and disseminate sustainable agri-food technologies, information and practices to serve objectives including poverty alleviation, income generation, food security and the sustainable use of natural resources. CIP was established in 1971 in Peru, which is part of potato’s center of origin and diversity, with an initial mandate on potato and expanding to include sweetpotato in 1986. Potato and sweetpotato are among the top 10 most consumed food staples globally and provide some of the most affordable sources of energy and vital nutrients. Sweetpotato plays a key role in securing food for many households in Africa and South Asia, while potato is important worldwide. Both crops grow in a range of conditions with relatively few inputs and simple agronomic techniques. Potato is adapted to the cooler environments, while sweetpotato grows well in hot climates, and hence, the two crops complement each other. Germplasm enhancement (pre-breeding), the development of new varieties and building capacity for breeding and variety testing in changing climates with emphasis on adaptation, resistance, nutritional quality and resource-use efficiency are CIP’s central activities with significant benefits to the poor. Investments in potato and sweetpotato breeding and allied disciplines at CIP have resulted in the release of many varieties some of which have had documented impact in the release countries. Partnership with diverse types of organizations has been key to the centers way of working toward improving livelihoods through crop production in the global South.

**Supplementary Information:**

The online version contains supplementary material available at 10.1007/s00122-023-04515-7.

## Potato and sweetpotato for the global south

The increase in demand for plant-based products by up to 70% forecasted for the next three to four decades cannot be met through increased production using current crop varieties and farming practices; and the magnitude of impact of climate change on crop yields is expected to be greatest in tropical regions of the global South (Voss-Fels et al. 2019; Challinor et al. 2014.) Potato (*Solanum tuberosum* L.) and sweetpotato (*Ipomoea batatas* L. Lam) are main resources for food security, employment and income in developing countries. They are among the top 10 most consumed food staples and provide some of the cheapest sources of energy and vital nutrients. While sweetpotato plays a key role in securing food for many households in Africa and South Asia (Fuglie 2007), potato is important worldwide; its short cropping cycle allows it to serve as a hunger-breaking crop and makes it suitable for intercropping and double cropping, especially in cereal-based production systems of Africa and Asia (Lutaladio and Castaidi 2009; Cromme et al. 2010). Both crops grow in marginal conditions with relatively few inputs and simple techniques– making them ideal “climate-smart” crops. Potato and sweetpotato production is steadily increasing in developing countries (Scott 2021).

Potato and sweetpotato are vegetatively propagated autopolyploids (2*n* = 4 × = 48 and 2*n* = 6 ×  = 90, respectively). While nearly all modern potato varieties are tetraploid, the Andean potato landrace groups Phureja and Stenotomum are still grown and appreciated for culinary quality near the crop’s center of domestication in Colombia, Ecuador and Peru are diploid (2*n* = 2 ×  = 24). The storage organs of potato are tubers, which are thickened stems with nodes or “eyes”, from which the crop is propagated. Sweetpotato storage organs are enlarged lateral reserve roots and propagation is by vine (stem) cuttings. Both crops are outcrossing and highly heterozygous; thus, their sexual/botanical seeds, which are referred to as true seed as distinct from the vegetative propagules, are each genetically unique recombinants. Outcrossing is promoted by self-incompatibility systems—gametophytic in potato and sporophytic in sweetpotato. The heterozygous, polyploid nature of potato and sweetpotato hampers the addition of new traits to existing varieties and slows down the assembly of favorable alleles by cross breeding. Potato has a notably low vegetative propagation rate of about 10 tubers per plant grown from one tuber or seed piece per crop season but produces about 200 botanical seed in each of its many fruits. Sweetpotato has a high vegetative propagation rate (about 30:1 vine cuttings per plant within 6 to 8 weeks), but produces only up to 4 true seeds per capsule. Potato tuberization is affected by photoperiod, and this response is genotype dependent. Potato tuberization is inhibited at high night temperatures (above 21 °C) that also often cause malformations. Sweetpotato on the other hand is widely adapted, especially to hot climates in the tropics and subtropics, although it is also grown in temperate climates.

## Evolving CIP’s vision for breeding

The International Potato Center (CIP) was established in 1971 under the inspiration of a group of researchers, governments and foundations who shared a common vision for a potato research and development institute in the crop’s center of origin and diversity. Aligned with the CGIAR (“Consultative Group on International Agricultural Research”), the initial focus of CIP was to develop the technologies needed to bring the increased yields of the green revolution to a broader set of crops and production environments. In 1986, the Center’s mandate expanded to sweetpotato. As a public breeding organization, CIP develops germplasm which is freely available to producers, researchers and other breeders, although material transfer agreements and IP regulations are in place. Its scope runs from ‘pre-breeding’, which bridges discovery research and applied crop breeding, to variety development, capacity building and knowledge exchange.

**Fig. 1:**
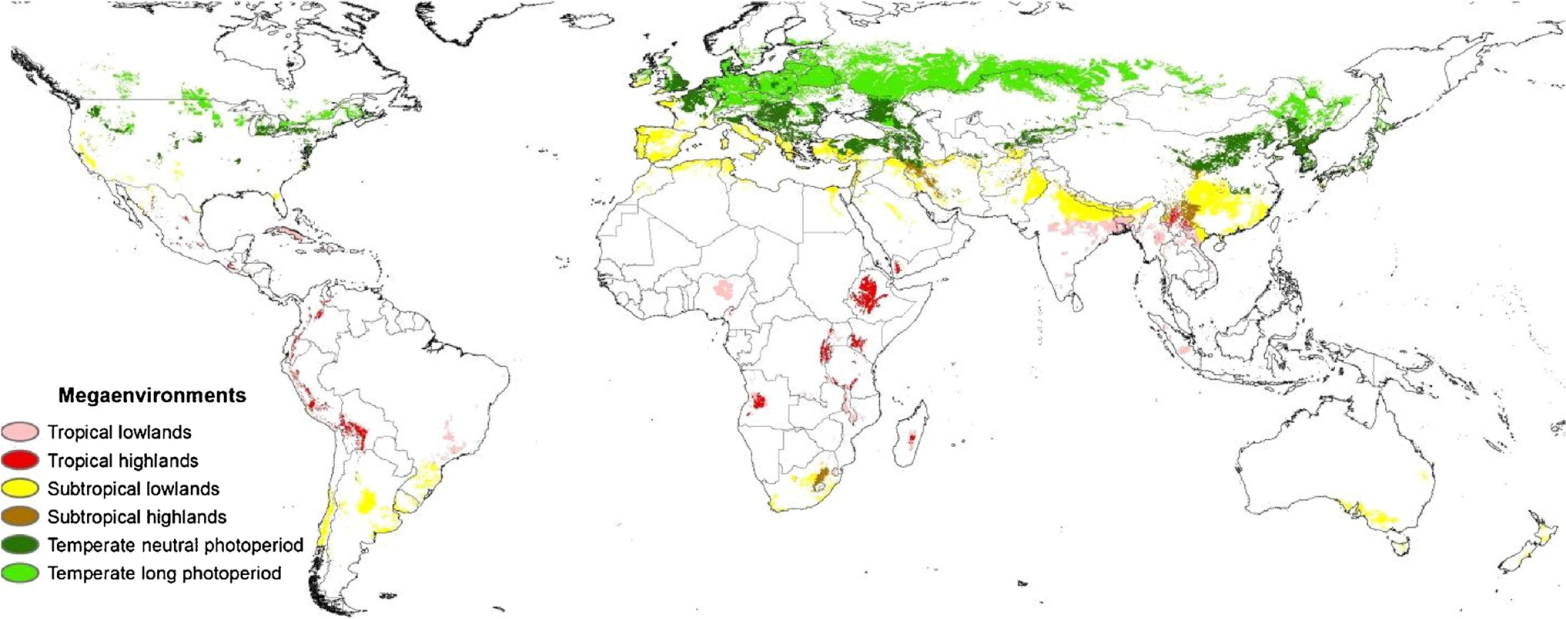
Potato mega-environments from Raymundo et al. 2018. Figure reproduced with the permission of the original publisher Elsevier (License number 5573241457253)

CIP’s breeding efforts began in the 1970s with the objective of improving potato resilience, production and consumption in the tropics. It was built on comprehensive collections of wild tuber-bearing “Solanum Section Petota” and cultivated landrace (*Stenotomum*, *Phureja* and *Andigenum* Group; Series Tuberosa) germplasm. Potato’s genetic resources range from diploid to hexaploid (Spooner et al. 2004), most of which are found in the equatorial Americas. Knowledge of reproductive barriers and means to overcome them make this vast reservoir of germplasm useful to breeders (Ortiz et al. 1997; Jansky 2006). By the late 1970s, and likely due to the global production of potato, seed tuber trade, and accompanying migration of pests and pathogens, potato breeding programs in the North had faced many of the same biotic constraints that would be met in the tropics, making their pre-bred genepools immediately useful as additional input germplasm for CIP. The Genebank at CIP (https://cipotato.org/genebankcip/), and the similarity of several Peruvian environments with other potato-growing regions targeted by CIP, make the Center’s location in Peru ideal for tropically oriented population improvement. Potato growing regions have been grouped into six mega-environments based on FAO climate classification (two each of temperate, subtropical and tropical environments) (Midmore et al. 1988; Raymundo et al. 2018) (Fig. 1). CIP addresses tropical lowlands, tropical highlands, subtropical lowlands and subtropical highlands. Some locations of Peru’s coast correspond to the same mega environment as the important south Asian winter potato production zone, and parts of the Peruvian Andes are similar to the East African and South Asian highlands, such that the recommendation of advanced clones bred in Peru has resulted in the selection of new improved varieties for local production and markets (Myrick et al. 2021). Early emphasis was on germplasm enhancement to transfer resistance traits from wild germplasm to sets of intermediate materials more useful in variety development, and diploid and interploid pre-breeding has continued to support tetraploid potato breeding at CIP (Bonierbale et al. 2020).

Through the late 1990s, potato breeding in Peru was complemented with hubs in the Philippines and Indonesia to identify and help address biotic pressures in warmer environments where multiple pests and pathogens challenge production. Additional postings of potato breeders in Cameroon and currently in Kenya, Vietnam and China have strengthened the largely centralized breeding strategy, in which advanced clones, progenitors and true seed progenies with superior genetic merit from each of CIP’s eco-geographically oriented advanced populations are developed in Peru and provided to National Agricultural Research Systems (NARS) as candidate varieties or breeding stocks. Feedback on performance from collaborating NARS and CIP researchers in the regions helps to inform breeding efforts in other environments.

Sweetpotato is of neotropical origin and according to the FAO data from 2021 cultivated in more than 110 countries from the equator to temperate climates with pronounced long hot summer seasons (https://www.fao.org/faostat/en/#data/QCL/visualize). In the 1980s, CIP’s sweetpotato germplasm acquisition focused on cultivated landraces, improved varieties and wild germplasm from seven Latin American countries. Among eight wild *lpomoea* species, diploid and tetraploid *I. trifida*—the likely ancestor of sweetpotato—were emphasized in CIP’s first experimental crossing with cultivated sweetpotato (Iwanaga et al. 1991; Freyre et al. 1991). Early population improvement was based on advanced germplasm introduced from the US as true seed tracing back to the Caribbean, and farmer’s varieties collected in South America, and targeted China and East Africa, with breeding hubs in Indonesia, Kenya and Peru. High dry matter (DM) and yield stability as required by farmers to increase their income through post-harvest processing, pest resistance and starch digestibility were CIP’s sweetpotato breeding objectives through the late 1980s. From the early 1990s, selection in the Amazon basin of Peru from large-scale polycrosses resulted in CIP’s first orange-fleshed sweetpotato (OFSP) population adapted to humid tropical climates and richer in beta-carotene (pro-vitamin A) than Africa’s predominant white and pale-fleshed types.

Evidence-based advocacy for the potential of OFSP to help combat vitamin A deficiency (VAD) among vulnerable populations (Low et al. 2007; Hotz et al. 2012) attracted significant donor interest for beta-carotene enrichment, which had been CIP’s top sweetpotato breeding priority with funding from HarvestPlus since 2003. Since Sweetpotato virus disease (SPVD) constrained the use of CIP’s advanced clones in East Africa, crossing blocks were established in African locations representing contrasting, high and low altitude, hot spot environments with year-round rainfall where the aphid transmitted potyvirus Sweetpotato feathery mottle virus (SPFMV) and the whitefly transmitted crinivirus Sweetpotato chlorotic stunt virus (SPCSV) interact to cause SPVD. Three sub-regional sweetpotato support hubs, located in Uganda, Mozambique and Ghana, were established in 2009 focusing on population improvement as a key part of CIP’s decentralized sweetpotato breeding strategy.

Breeding of both CIP crops has been carried out in the context of interdisciplinary programs that seek integrated solutions and provide guidance to orient breeding. For example, the perishable nature of potato, sweetpotato and their vegetative seed/vines call for an alliance between plant breeders, physiologists and agronomists who help build knowledge and capacity to evaluate candidate varieties along with on farm research that identify storage traits, tuber seed storage techniques and vine survival systems compatible with local, low input conditions (e.g., Carli et al. 2010). Further engaging pathologists, economists and crop modelers, biophysical conditions, local practices, and global economic information as well as performance simulation are applied to the setting of priorities for investment in breeding for specific traits and the recommendation of screening sites and varieties for target regions (Forbes et al. 1997; Pérez et al. 2001, Gastelo et al. 2014; Hareau et al. 2014; Raymundo et al. 2018, Grüneberg et al. 2015; Ojwang et al. 2023). While CIP does not engage directly in commercialization and distribution of its products, the breeding program counts on complementary specialists in seed production to innovate and collaborate on strengthening value chains for effective delivery of appropriate technologies. For example, the USAID-supported three‐generation seed production model (3G) project led by CIP seed and crop management specialists strengthened capacities and leveraged the comparative advantages of myriad public sector agencies, private firms and non-profit companies in Kenya, Rwanda and Uganda to raise awareness and implement best practices for improved access to quality seed of popular and new potato varieties. The CRP RTB developed a seed systems toolbox (Andrade-Piedra et al. 2022), and the current CGIAR portfolio includes the Seed Equal Initiative that focuses on seed delivery with the goal of increasing the genetic gain in farmers’ fields.

## Partnerships

Through the late 1980s, a network of advanced research institutions (ARI) and NARS was established to expand the range of germplasm, environments and capacities available to the global effort of improving potato for the tropics. Research contracts with universities in North America and Europe provided expertise and training for developing country researchers and helped understand cross ability; incorporate novel traits from wild relatives into cultivated potato; and develop effective strategies for germplasm enhancement.

**Table:** 

Early research contracts jump start potato breeding at CIP	
Cornell University’s interdisciplinary potato improvement team provided an excellent model for building institutional capacity. From 1972 until 1992, CIP helped fund the training of 8 South American, one African, one Asian and three US professionals in the context of research on breeding with Neotuberosum—*S. tuberosum* group Andigenum germplasm bred for adaptation to long daylength – and *S. berthaultii* with complex insect resistance, to broaden the genetic base of commercial potato in warm, long-day environments (Plaisted et al. 1992, 2019). They addressed heat, heterosis for yield, market traits and resistance to PVY, PVX, late blight (*P. infestans*), scab (*S. scabies*), green peach aphids (*M. persicae*), Colorado potato beetle (*L. decemlineata*) and cyst and root knot nematodes (*Globodera* and *Meloidogyne spp*). Midway into this program, CIP provided germplasm bred from Andigenum and *S. vernii* that enabled Cornell ‘s precautionary breeding against the threat of *Globodera pallida* (the pale cyst nematode; PCN) from South America. CIP’s bred germplasm was introduced as true seed, and in each generation, tuber families from pollination with neotuberosum x tuberosum hybrids that were immune to PVX and PVY, resistant to *G. rostochiensis* R1A (golden nematode, (GN)), resistant to late blight, and adapted to long-day growing conditions were sent to Peru where screening for races 4A and P5A of *G. pallida* could be performed (Brodie et al. 1991). Subsequent backcrossing with New York states GN resistant varieties overcame the extensive vine growth and late tuberization of selections with the added resistance (Silvestre et al. 2021). The resulting adapted germplasm was provided to additional breeding programs for which it would considerably shorten the time required to develop commercial varieties with resistance to *G. pallida*. The contracted research further extended to molecular diversity assessment and genetic mapping in the ‘80’s, leading to the first molecular map of potato, demonstrating homeology with tomato and opening the way for the power of comparative genomics (Bonierbale et al. 1988)	

**Fig. 2:**
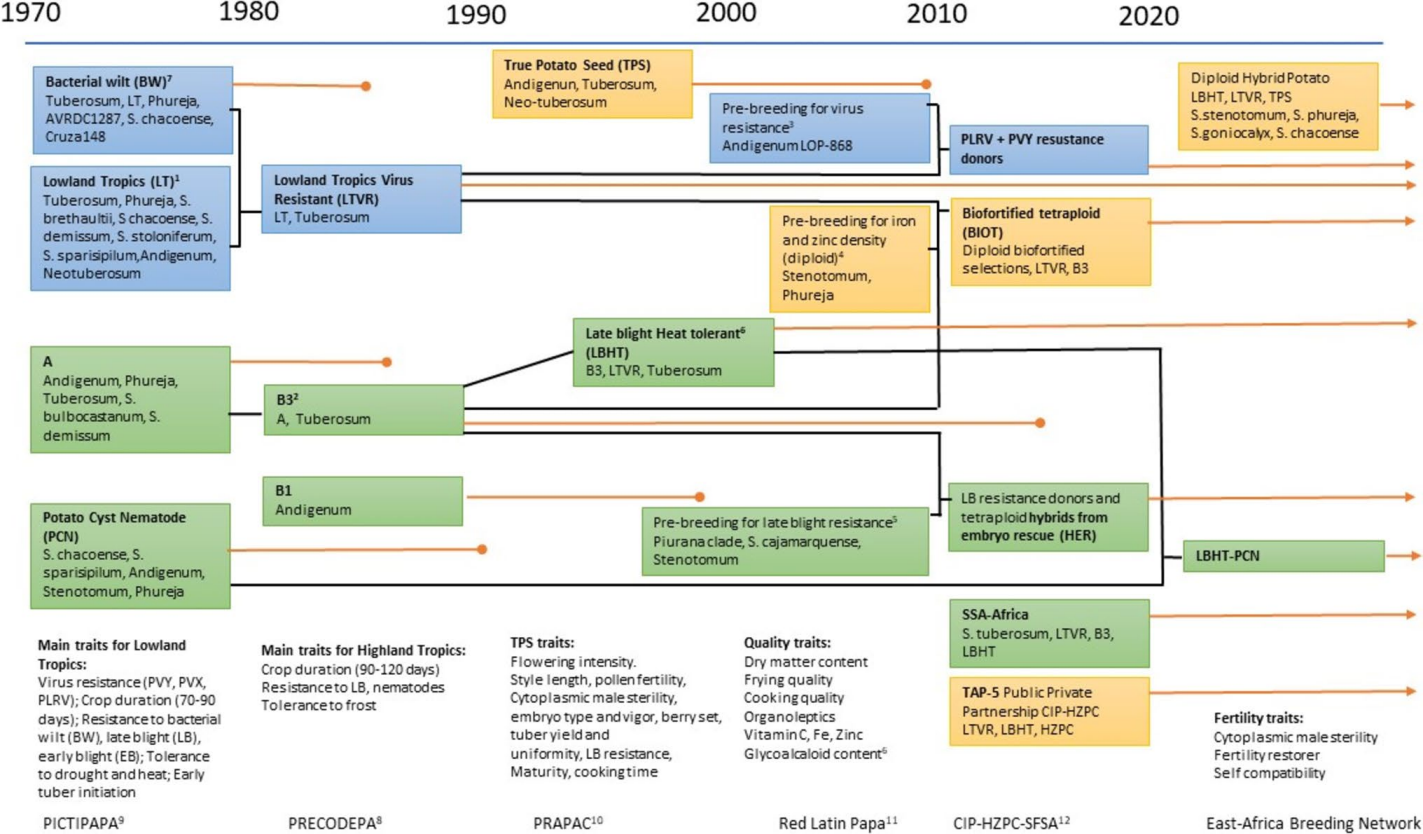
Approximate timeline of target traits and trait sources in CIP’s potato breeding program. The breeding pipelines are organized based on broad target agroecology: highland tropics (light green boxes) and lowland tropics (light blue boxes). Yellow boxes represent pipelines targeting both agroecologies. Black lines depict how germplasm from pipelines were combined. Orange lines ending in circle show the end point of the pipelines while the orange lines ending with an arrowhead depict pipelines that continue. ^1^Reynolds and Ewing (1989), Watanabe et al. (1994), Watanabe et al. (1995), Mendoza and Haynes (1974, 1976), Mendoza, (1989), ^2^Li et al. (2012), Lindqvist-Kreuze et al. (2014), Lindqvist-Kreuze et al. (2021), Jiang et al. (2018), ^3^Mihovilovich et al. (2007), Velásquez et al. (2007), ^4^Amoros et al. (2020), ^5^ Pérez et al. (2001), Trognitz et al. (2001), Pérez et al. (2022), Villamon et al. (2005), Lindqvist-Kreuze et al. (2010), Ordoñez et al. (2023), ^6^Benavides et al. (2017), Muñoa et al. (2022), ^7^Ciampi and Sequeira, (1980), French and Lindo (1982), Priou et al. (2005), Gutarra et al. (2015), ^8^Niederhauser and Villarreal (1986), ^9^Pictipapa (1995), ^10^Mendoza et al. (1994), ^11^https://www.scribd.com/document/72800341/Red-LatinPapa-Red-Iberoamericana-de-Innovacion-en-Mejoramiento-y-Diseminacion-de-la-Papa#, ^12^Sharma et al. (2020)

Research agreements were complemented by multi-institutional regional networks (Fig. 2) to promote the development and distribution of genetic materials, practices, information and experiences, and effectively integrate the key public and private sector players that define the supply and demand for new potato varieties. Details on research networking, particularly involving Japan and Indonesia, during the early years of CIP were recently summarized by Watanabe et al. (2021).

Early partnerships for sweetpotato breeding were of a different nature due to its importance as a staple, food security crop with significant landrace germplasm and distinct quality preferences in the Americas, Asia and Africa by the time the CGIAR was established. With respect to breeding, however, it was a neglected crop with scarce reports of objectives and methods (Kobayashi and Miyazaki 1977), except in the USA at North Carolina State University (NCSU) and in China which in the 1970s produced and utilized about 80 percent of the world's sweetpotato. By the late 1980s, two research agreements had been established with institutions in China, one at Xuzhou Sweetpotato Research Center (XSPRC) and the other at Guangdong Academy of Agricultural Science (GAAS), to catalyze the evaluation of Chinese sweetpotato collections for characters important in removing major constraints to production and use.

Sweetpotato breeding has also been advanced through regional networks and consortia, from the Asian Network on Sweet Potato Genetic Resources (ANSWER) and CIP’s leadership of the Vitamin A for Africa (VITAA) Consortium, and the multi-partner, multi-donor initiative Sweetpotato for Profit and Health Initiative (SPHI) which reached 6.8 million African households in 15 target countries with improved varieties, promoting their diversified use.

Partnerships with non-governmental organizations (NGOs) have been most valuable for their direct involvement with farmers at the local level, allowing them to voice their needs and assess new varieties and concepts in the context of farming systems and livelihoods. Extending collaboration to wider consortia of local institutions including schools has been valuable for awareness raising and continuity of CIPs efforts toward variety and behavior change, the latter being particularly relevant to the introduction of more nutritious, orange-fleshed sweetpotato to Sub-Saharan Africa, where white-fleshed varieties were traditionally preferred.

Collaboration with China has been significant since the establishment of CCCAP (CIP China Center for Asia and Pacific) and deepened further after the establishment of the new research facility in Yanqing, Beijing in 2017 (https://cipotato.org/pressreleases/new-cccap-facilities-china-mark-new-era-root-tuber-research-asia/).

Since 2015, with the support of the Syngenta Foundation for Sustainable Agriculture (SFSA), CIP has partnered with the leading global potato seed company Royal HZPC Group to breed short cycle, disease resistant varieties for fresh and processing markets of Southeast Asia and beyond. The project is an example of combining complementary gene pools: on one side genetic progress at HZPC for globally relevant table and processing market traits, and on the other CIP’s relevant traits for adaptation, climate resilience and disease resistance (Sharma et al. 2020). A unique feature of the SFSA/HZPC/CIP partnership is that part of the profits from joint commercialization of potato varieties will flow to the International Treaty for Plant Genetic Resources and CIP’s breeding program. Along with other partnerships with private seed interests, this is an important part of CIPs strategy to support the complete value chain from breeding through marketing its products for delivery to the field.

## Target traits

### Tropical adaptation

CIP has taken a population breeding approach to the accumulation of resistance to biotic and abiotic stress in broadly based gene pools agronomically adapted to tropical highland and lowland potato production environments. CIP’s major objectives for the highland tropics have been the drastic reduction of the crop duration from 5–6 months to between 90 and 100 days and increasing levels of resistance to late blight, while frost and cyst nematode resistance breeding were taken up sporadically (Fig. 2). In the absence of resistant varieties, potato late blight is controlled with frequent fungicide applications, and where these are not used, complete crop loss is not uncommon (Haverkort 2009). Each of the new approaches that promise to facilitate the incorporation of new genes into potato varieties, including transgenic modification as achieved by CIP and others (e.g., Ghislain et al. 2019; Byarugaba et al. 2021) and true hybrid breeding at the diploid level (Su et al. 2020), is foremost being applied to develop durable resistance to this disease by stacking combinations of the many late blight resistance R genes that have been identified and isolated to date (Paluchowska et al. 2022). Meanwhile, the seasons and sites that are suitable to potato production in the lowland tropics (< 1,500 masl) present air temperatures above the ideal of 18–25 °C for above-ground mass growth and soil temperatures above the optimum of 7–19 °C for tuber growth, and pressure from viruses, bacteria, and pests. Potato virus Y, (PVY), potato virus X (PVX) and potato Leaf Roll Virus (PLRV) are of prime importance in warm climates with high vector populations, causing high net losses due to reduced yield, tuber defects and downgrading of seed crops (Solomon-Blackburn and Barker 2001). Increasing the frequencies of major gene, extreme resistance to the former two, and levels of quantitative resistance to the latter underlie CIP’s lowland tropic population improvement, including discovery, characterization and cloning of a new source of PLRV resistance from Andigena germplasm, the latter in collaboration with the Sainsbury Laboratories at the John Innes Centre (United Kingdom) (Velásquez et al. 2007; Mihovilovich et al. 2007; Heal et al. 2022). Interestingly, although the source landrace “Alca Tarma” was collected in the high Andes, where virus vector populations are non-existent, the resistance limits both infection and multiplication of the virus. *Rladg* is homologous to the *Bs4* gene which contributes to a hypersensitive response (HR) in tomato by mediating recognition of avrBs4-expressing strains of the bacterial spot pathogen *Xanthomonas campestris* pv. *vesicatoria* (Heal et al. 2022) pointing to conservation and broad function as opposed to acquisition or evolution in the potato clade of Solanum.

Andigenum germplasm is short-day dependent for tuberization, while tuberosum germplasm is able to tuberize in a broader photoperiod regime, including long-day conditions (photoperiod of over 12 h) (Rodríguez-Falcón et al. 2006). Tuber development is coordinated with vine maturity which is explained by a large effect QTL in chromosome 5 (Visker et al. 2003) and contains the gene CDF1 (Kloosterman et al. 2013). The different allelic variants of this gene control the circadian rhythm-dependent tuberization response. While the andigenum potatoes carry the allele StCDF1.1, potatoes that are early maturing and tuberize under long days, carry variants StCDF1.2 and 1.3 that encode truncated proteins resulting in the blocking of the signal that would normally delay tuberization (Kloosterman et al. 2013; Morris et al. 2014). Breeding for early tuber initiation and bulking (Mihovilovich et al. 2014) has been possible under short days, combining Andigenum-derived germplasms capacity to effect complete crop cover, and maintain high bulking rates, with tuberosum germplasm. Tuberosum and Neo-tuberosum (Bradshaw 2009) respond to short days by a hastening of tuberization, accompanied by foliar senescence before a full crop can be produced. The combination of andigenum and tuberosum has been key to wide adaptation and harvest can be practiced at 70 days after planting with CIP-bred potato varieties in the subtropical lowlands of Asia (Devaux et al. 2014). For example, despite the often-reported negative correlation between late blight resistance and earliness of potato, CIP’s tropical highland, late blight resistant selection CIP393371.58 (released as Chucmarina in Peru, Kenya MPYA in Kenya, Belete in Ethiopia and BARI Alu-46 in Bangladesh, yields well at 70 or even 65 days after planting in subtropical lowland conditions, or may be grown as a mid-season crop for higher yields at 90 days according to cropping patterns (Mahmud et al. 2022). Evaluation of samples of the lowland tropic virus resistant population under subtropical and continental temperate conditions in Uzbekistan and Tajikistan identified day neutral clones and enabled the release of CIP-bred virus resistant potato varieties including Sarnav/Fayzobod (CIP397077.16), Pskom (CIP390478.9) and Tajikiston (UNICA; CIP392797.22) that provide high yields in 90 days under the hot, dry, saline conditions of the Aral Sea (CIP 2015) and in 70 days in the tropics.

For sweetpotato, CIP’s germplasm screening for climatic and location effects on storage root thickening for starch accumulation in reserve roots began in the high and low jungle regions of Peru. Screening and breeding for pest and disease resistance has addressed nematodes (*Meloidogyne* spp. and SPVD in hot humid tropical climates) and sweetpotato weevil (*C. foricarius)* in dry subtropical climates. Resistance or tolerance to these segregates in most breeding populations. Intensive collaboration was established with Chinese breeders to whom seed families with high dry matter content and resistance to root-knot nematode, and with high dry matter and wide adaptation were sent from Peru, Nairobi and Bogor, respectively, in the early 1990s. Advanced clones from CIP-Bogor’s modified shuttle breeding program with Japan, selected at a humid tropical site with poor soil, a cool highland, and a mid-elevation site with fertile soil, contributed to adapting early sweetpotato populations to diverse growing conditions of Asia (Ma et al. 1998). Shuttle breeding traditionally refers to the evaluation of two or more filial generations (e.g., F2 and F3) of a population in contrasting environments in one year to advance it and shorten the breeding cycle of inbred crops like rice and wheat for diverse constraints and adaptation (Anon, 1989; Alahmad et al. 2022). Modifications for sweetpotato and potato have utilized early clonal generation (F1 true seed or tuber family) performance data from multiple strategic locations to inform crossing and required more than one year due to longer growing cycles, but the approach has likewise contributed to efficient breeding for new and different environments.

Drought avoidance resulting from deep rooting of both crops was identified by CIP physiologists in the late 1980s (Ekanayake et al. 1989), and escape by deep rooting and early maturity are selection criteria in CIP’s drought tolerant sweetpotato breeding strategy (Low et al. 2020). Evaluation and selection of sweetpotato and potato for drought tolerance were strengthened in the early 2000s to identify integral as well as indirect selection measures for maintenance of yield under low water availability and develop understanding of GxE and drought tolerance mechanisms that guide breeding strategies (Schafleitner et al. 2007; Cabello et al. 2013; Andrade et al. 2016). For example, the reflectance measures ‘vegetation index’ and ‘normalized difference vegetation index’ were shown to be significantly higher in tolerant potato genotypes and correlated with yield under drought (Schafleitner et al. 2007). A significant link between plant vigor and metabolite fumarate was identified under drought in a potato association panel (Toubiana et al. 2020). In sweetpotato association panel candidate genes and genome regions associated with the complex trait ‘continuous storage root formation and bulking’ during drought were identified (Bararyenya et al. 2020). GCA was found to be more important than SCA for growth, physiological and tuber yield-related traits under drought in potato, and the best families were used to advance local breeding for drought tolerance combined with late blight resistance in Ethiopia (Hirut et al. 2017).

### Table and processing quality

The predominant consumption of potato as a fresh crop in the global South, rather than as a processed one, has influenced CIP’s breeding priorities. Stable, high tuber dry matter (> 18%), tuber size, uniformity and appearance including shape and shallow eye depth, freedom from off flavors, e.g., due to glycoalkaloid accumulation (Benavides et al. 2017) and low weight loss under storage have been the primary potato quality traits under selection at CIP, while cooking type, low reducing sugars and light chip color are taken as secondary traits. Elicitation of farmers’ and consumers’ appreciation among panels of bred and landrace clones has shown cooking time, appearance after boiling, taste, texture, and storability as well as yield and resistances to influence varietal preferences in Ethiopia, Kenya, Uganda and Peru (Tesfaye 2013; Asfaw et al. 2018; Scurrah et al. 2019; Mudege et al. 2021). With a view to enhancing the adoption of new varieties primarily bred for agronomic traits, participatory variety selection is used in taking advanced clones forward in variety release, and, iteratively, to identify the most promising (best bet) sets of advanced clones to offer to particular collaborating NARS or NGOs who engage locally with end-users (primarily farmers). To complement evaluation for table quality, CIP has characterized samples of landrace and improved populations for relationships among nutritional quality traits—vitamin C, carotenoids, anthocyanins and phenolic compounds—that influence the bioavailability of iron and zinc in addition to their respective health properties (Burgos et al. 2009a, 2009b, 2013) these latter which were targeted in biofortification breeding (see Iron and Zinc Potato, below). Knowledge of genetic relationships and dietary interactions among these nutritional and anti-nutritional compounds guides their consideration in CIP’s ‘biofortified potato’ breeding pipeline (Andre et al. 2015; Burgos et al. 2020).

Genetic studies on components of variation for DM accumulation and processing quality of potato in cool and warm environments (Amoros et al. 2001), projects on chips/French Fries (FONTAGRO; PepsiCo), and collaboration with McCain under the Red LatinPapa network enabled the identification of superior progenitors and elite clones for processing. Fripapa was one of the first CIP-bred varieties released for processing as crisps (Ecuador), and among other CIP-bred varieties, Victoria (Kenya, Uganda), UNICA (Peru, China, Bhutan, India) and Nova (CIP395186.6), released in 2009 and CIP-BACATA (CIP392759.1), released in 2022 in Colombia have gained appreciable percentages in the processing industry or have just been released. In accord with demand from national programs, CIP has increased its priority on potato processing quality, and regular assessment as well as greater weight has been placed on organoleptics and determination of cooking type through the 2000s. Growing consumer income levels, changing rural and urban infrastructure, lower potato prices and greater numbers of fast-food restaurants are the main factors increasing the demand for processed potato in developing countries. NARS increasingly include processing quality in their assessment of CIP-bred potato for variety release (e.g., Degebasa et al. 2021; Rukundo et al. 2022). Three potato varieties suitable for oven-baking and oil-frying were released in Peru in 2023 (Gastelo et al. 2023). In this case, the final selection was made in collaboration with local restaurants upon CIP Breeders’ advice based on chipping tests of elite clones bred primarily for disease resistance and yield.

Sweetpotato quality traits related to texture of cooked storage roots are critical characteristics which—along with sweetness and flesh color—largely determine consumer preference. The strength of orange color of sweetpotato flesh is positively correlated with contents of beta-carotene, a precursor of vitamin A, and negatively correlated with dry matter, a major component of preferred texture. Where pest and disease pressure is moderate, there are large opportunities to improve quality traits. Three major quality classes are recognized for fresh consumption: ‘dessert types’ with deep orange flesh color (high in beta-carotene), low dry matter content (< 28%), moist texture and high flavor impact due to sweetness and aroma.; ‘staple types’ which have high dry matter (> 28%), typically white flesh lacking b-carotene, with drier texture, and lower flavor impact (Martin and Jones 1986; Kays et al. 2005); and ‘orange-fleshed sweetpotato (OFSP)’ dry and starchy types’ with pale to medium orange flesh color and medium to high dry matter with pronounced dry texture and moderate flavor impact. The latter type is found among landraces in Africa (Tumwegamire et al. 2011), and along the arid Pacific coast of Latin America. CIP and collaborating sweetpotato breeders have applied tools of the CGIAR Gender in Breeding (GBI) Initiative to distinguish gender preferences for the quality of boiled or steamed sweetpotato in Uganda (Polar et al. 2022). Studies revealed that men and women had different quality preferences for boiled/steamed sweetpotato, driven by gender norms and roles (Mwanga et al. 2020). Recently, a lexicon, protocol and a scoring system were developed to facilitate the selection of priority sensory attributes for consumer preference in sweetpotato during the breeding process (Nakitto et al. 2022, 2023). These user’s preferences have been included in the current sweetpotato target product profiles, which now incorporate quality traits such as mealiness, sweetness and cooking time as essential traits for breeding.

### Beta-carotene

The consumption of foods which contain beta-carotene, a precursor to vitamin A, can mitigate Vitamin A deficiency (VAD) (Adekambi et al. 2023). VAD affects over 200 million women and children worldwide (WHO 2009) and can lead to weakened immune system, growth limitations, blindness and increased mortality (Sommer and West 1996). Beta-carotene-rich OFSP has been part of CIP’s breeding portfolio since the early 2000s thanks to the interest for OFSP adapted to East African growing conditions and consumer needs. Despite initially limited investments strong knowledge exchange was established with two major achievements—breeders made many crosses via poly-crosses and some rare local OFSP landraces were found—up to that time it was assumed that the sweetpotato diversity in Africa is white- or cream-fleshed. These African OFSP became parents in many breeding programs of Sub-Saharan Africa, and by 2008 varieties had surpassed the target level of 125 ppm of beta-carotene-prompting HarvestPlus to label the product as biofortified (Gruneberg et al. 2015; Moumoni Koala et al. 2013). OFSP provides 50% of the RDA for vitamin A in populations of Asia and East- and Southern Africa with high sweetpotato consumption (daily intake of > 80 g per capita).

### Iron and zinc

About half of children under five years of age in Sub-Saharan Africa (SSA) suffer from iron (Fe) deficiency, the major cause of anemia and the most common micronutrient deficiency globally, which in infancy and adolescence can lead to impaired mental development (Fretham et al. 2011). Zinc (Zn) deficiency contributes to decreased immunity and increases the risk of stunting, common childhood infections and mortality (Roohani et al. 2013). CIP’s breeding program has sought to increase mineral density of both potato and sweetpotato and in parallel has been collaborating on studies of the bioavailability of iron and zinc in the human diet.

For potato, CIP identified sources of elevated iron and zinc contents in diploid landrace groups Phureja and *Stenotomum* (Burgos et al. 2007) and utilized these in breeding. Recurrent selection at the diploid level revealed high heritability (0.81 for both iron and zinc) and high positive correlation enabling genetic gains of more than 29% iron and 26% zinc (Amoros et al. 2020). Selected iron‐ and zinc‐dense genotypes with high, positive general combining ability among the diploid biofortified population were identified for use in an interploidy (4x–2x) breeding scheme. After one cycle of recombination, the micronutrient content in the new tetraploid hybrids was significantly higher than in the modern clones, indicating that the trait can be incorporated into advanced populations. Field and laboratory evaluations conducted from 2018 to 2022 resulted in the identification of three biofortified clones for potential variety release. These are first-generation biofortified tetraploid potato with iron content of 0.40–0.49 mg/100 g of fresh weight (FW). This is on average 50% higher than in current modern varieties grown in Peru (0.3 mg/100 g FW). The tetraploid hybrids have been also tested in Rwanda, where promising candidates suitable for local conditions were identified (Rukundo et al. 2023).

While iron concentrations in potato are lower than those reported for cereals and legumes, potato iron bioavailability has proved to be significantly higher due to the presence of high levels of vitamin C, which promotes iron absorption in the human body, combined with low levels of phytic acid, an inhibitor of iron and zinc absorption. Yellow-fleshed potato bred at CIP for high Fe content had a remarkably high level of bioavailable iron ranging from 16% in Peruvian women with moderate iron stores (Burgos et al. 2023) to 29% in Peruvian women with low iron stores (Jongstra et al. 2020). Sweetpotato iron bioavailability has been reported between 5 and 8% in women from Malawi (Jongstra et al. 2020). A recent study has reported zinc absorption levels of 20–25% in potato (G. Burgos, personal communication).

Sweetpotato storage roots exhibit a beneficial correlation between the micro-nutrients b-carotene, iron and zinc contents (Grüneberg et al. 2015), making them an ideal candidate for double or triple biofortification. In a single reciprocal recurrent selection cycle, genetic gain of 20% for iron was demonstrated in an OFSP breeding population (Grüneberg et al. 2022). In this population, the mean level of iron reached 0.72 mg/100 g fresh weight, and the best clone had 1.47 mg/100 g iron associated with 0.95 mg/100 g zinc. The target levels to reach 50% of the recommended daily availability that is required from a biofortified crop are 1.8 mg/100 g iron and 1.2 mg/100 g of zinc, assuming an iron bioavailability of 5% (Jongstra et al. 2020).

Both iron-biofortified potato and sweetpotato have the potential to contribute to reduced iron deficiency, particularly among people who consume these as a staple crop. The consumption of 500 g of iron-biofortified potato provides 27% of the iron requirement of women in the Peruvian highlands with moderate iron stores (plasma ferritin, pf, around 20 µg/L) and will provide more than 50% of the requirement for women with low iron stores (pf around 10 µg/L). In a similar way, consumption of 400 g of iron-biofortified sweetpotato by women in Malawi can contribute 18% of the daily iron requirement of women with moderate stores and 30% of the requirement of women with low iron stores.

### Market segments, breeding pipelines and product profiles

The Excellence in Breeding Platform (EiB; (https://excellenceinbreeding.org/CtEH)) that is currently part of the Accelerated Breeding Initiative of One CGIAR is working with CGIAR and NARS breeding programs to prioritize and focus their breeding efforts for maximum return on investment, increased genetic gains and impact on people in low- and middle-income countries. One of its premises is that more precise description of the several market segments breeding programs address, better definition of the specific sets of key traits (“product profiles”) required in the ideal product for each market segment, and alignment of corresponding breeding pipelines to develop those products will be more efficient than breeding schemes that might confound the needs of different consumers and producers. The segmentation considers the geographical region, agro-ecological zone(s), end use (table, processing, feed), color (skin, flesh), production environment, production system (rainfed/irrigated), and maturity. Product profiles have been designed for each of the market segments and consist of a set of traits relating to root/tuber appearance, processing, consumption (flavor, texture, cooking quality, cooking time, glycoalkaloid), nutritional value (beta-carotene, anthocyanin, iron, zinc), yield, maturity, and disease resistance. Each trait has been classified as “essential” or “nice to have” and assigned a minimum or maximum level. Desirable yield is determined in relation to a dominant variety in the market segment. The current CIP breeding pipelines and market segments have been thoroughly described in detail by Ojwang et al. (2023).

**Fig. 3:**
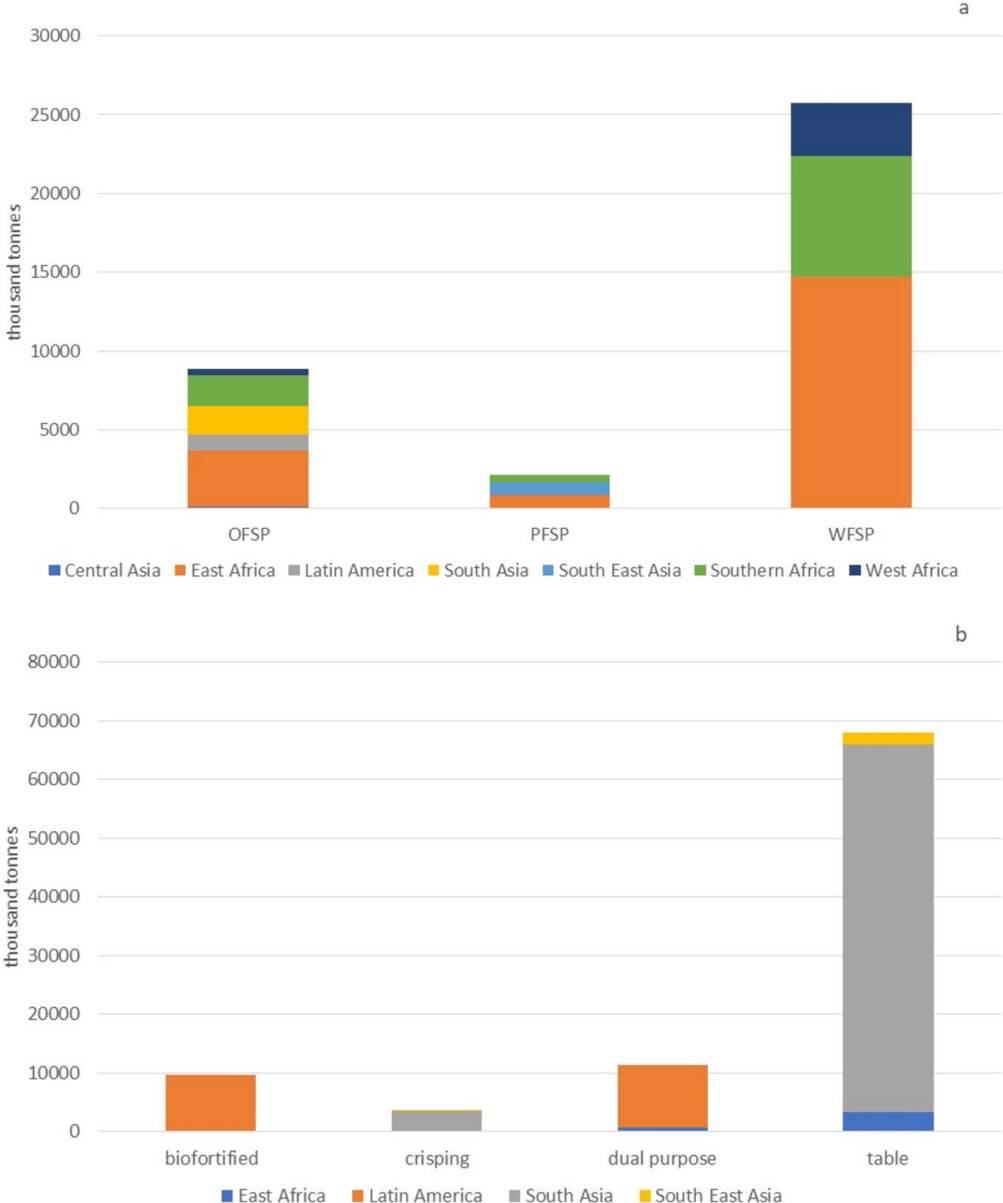
Production of sweetpotato **A** and potato **B** in the One CGIAR regions, excluding China, aggregated based on the main usage types. OFSP = orange-fleshed sweetpotato, PFSP = purple-fleshed sweetpotato, WFSP = white-fleshed sweetpotato

CIP’s sweetpotato breeding pipelines are implemented by three major breeding hubs in Uganda, Mozambique and Peru–the latter linked to variety development in Bangladesh. These breeding hubs currently address about 3.5 M hectares and 37 M tonnes in 30 countries across seven CGIAR regions (Grüneberg et al. 2022; Ojwang et al. 2023) (Fig. 3A, Table 1).

**Table 1:** The main breeding products of sweetpotato and potato and the global CIP breeding hubs

Main breeding products	Breeding hub
*Sweetpotato*	
OFSP for wide adaptation and earliness with a very short crop duration	Peru, Bangladesh
OFSP and WFSP with high DM content and strong resistance to sweetpotato virus disease (SPVD) for East Africa	Uganda
OFSP and WFSP with medium–high DM for drought-prone areas	Mozambique
OFSP double biofortified with high iron	Peru
PFSP with high DM for fresh consumption and processing use	Peru
*Potato*	
Highland and mid-elevation potato with late blight resistance	Peru
Subtropical early maturing lowland table and processing potato with virus resistance	Peru
Biofortified table potato	Peru
Asian table and processing potato for tropical and subtropical areas	Vietnam
East- African table and processing potato for tropical highlands	Kenya
Pre-breeding populations for various agroecologies	China
Table and processing potato for temperate and subtropical China	China

*OFSP* Orange-fleshed sweetpotato, *WFSP* White-fleshed sweetpotato, *PFSP* Purple-fleshed sweetpotato

CIP’s potato breeding pipelines currently target two major eco-geographies: tropical highlands and subtropical lowlands, across South Asia, Southeast Asia, East Africa, and Latin America and the Caribbean, corresponding to 5 million hectares and 93 M tonnes in 14 countries, excluding China (Fig. 3B, Table 1).

## Breeding approaches

### Breeding potato as a true seed crop

The term true potato seed (TPS) originated in the 70’s to describe a potato crop grown from botanical seed produced by crossing heterozygous potato parents. Among root and tuber / clonal crops, potato’s prolific botanical seed production and consequent large family size make it uniquely suited for this approach. The premise was that production of potato from botanical seed would reduce the cost of and increase availability of planting material, reduce seed storage losses and disease load, and enable more flexible planting times. Breeders explored a range of 4x–2x, 2x–2x, and 4x–4x hybrid and open-pollinated breeding schemes to develop heterogeneous, tetraploid TPS varieties. CIP’s TPS breeding addressed reproductive traits like flowering intensity, style length, fertility, berry set and seed size/ weight and vigor in addition to yield, late blight resistance and uniformity of tuber appearance, maturity and cooking time. The heterogeneous constitution of TPS, with several *R* genes dispersed among the progeny to be grown in the field, was proposed to help slow the spread of late blight.

One step used by CIP to accelerate the development of tetraploid TPS parents was to haploidize and allow the redoubling of selected lines in tissue culture. This allowed partial inbreeding and high selection intensity, purged some negative alleles, and increased the frequency of traits like red skin color, *R* gene resistance and fertility restoration. Research investigated the utility and inheritance of tetrad sterility-type cytoplasmic male sterility (CMS) from asymmetric somatic hybridization to develop male sterile female parents for ease of seed production. For this, organelles from a CIP-bred line with PVY resistance from *S. stoloniferum* and from *S. verrucosum* were transmitted into the protoplasts of fertile potato genotypes in collaboration with the Weizmann Institute of Science in Israel (Golmirzaie et al. 2003).

High heritability for plant vigor and tuber uniformity were found in CIP’s TPS population (Golmirzaie 1985). Progeny of tetraploid progenitors that flower well and set berries under warm tropical conditions were evaluated in genetic designs (North Carolina experimental Design I) under short days and high temperatures of San Ramon and contrasting cooler conditions of Lima, Peru. Superior TPS parental lines were selected for GCA for tuber yield, earliness, color, uniformity, and shape (Golmirzaie et al.^1994^).

Collaboration with NARS such as INIA (Chile) and CPRI (India) were established for the bulk production of botanical seed of TPS varieties, which were tested in target countries including Nepal, Vietnam, Egypt, Uzbekistan and China. However, experimentation, release and adoption of TPS varieties by farmers showed that the advantages of using TPS only translated into economic benefits as compared to tuber seed when the latter was costly or not available (Almekinders et al. 2009). Despite its promise as an alternative seed material in non-traditional areas, the uptake of TPS was limited in the absence of appropriate partnerships to develop technologies to produce seedling tubers, or treatments to allow direct planting of the small seed. CIP discontinued TPS breeding in 2007, until such time as investment might be made from the private sector to fill out the research/ development pipeline and value chain. Nevertheless, knowledge and experience from TPS breeding are useful to renewed efforts in potato production from true seed from inbred diploid parents (Lindhout et al. 2011); CIP’s TPS progenitors are valuable genetic stocks due to their reproductive traits. As females, several are male sterile and produce exceptionally large berries, while males and females have high combining ability for desirable tuber traits.

### Accelerated breeding scheme

One of CIP’s major steps forward for more efficient population improvement was the accelerated breeding scheme (ABS) first applied in Peru for sweetpotato in 2004 to reduce breeding cycle length (Grüneberg et al. 2009; Lebot 2010). ABS targets early breeding stages and replaces temporal by spatial variation of test environments. It is built on the fact that in clonally propagated crops, each true seed plant is already a potential variety. Cycle length is reduced when each genotype is evaluated in small, unreplicated plots at two or more locations in the first year. This is theoretically efficient as long as genotype by season interaction is small when compared to other variance components of genotype by environment interaction.

The ABS facilitates the rapid entry of new clones into advanced trials. With 30 or more sweetpotato vine cuttings per plant many 1 m row plots can easily be established at several locations within 3 to 4 months and used to estimate breeding values. The resources required are manageable if controlled cross breeding is applied with family sizes between 10–20 genotypes. Furthermore, the linking of population improvement with variety development is straightforward (Diaz et al. 2021; Grüneberg et al. 2022). However, application of ABS to elite cross introductions handling 10 k genotypes and more might require modifications—for example by using a modified independent culling selection in combination with index selection (Elston 1963) using Spanakakis’s approach to aggressively discard or even not harvest genotypes that have not reached standards (Diaz et al. 2023).

ABS has been less straightforward in potato due to its lower vegetative propagation rate. It is practical, however, when sets of tuber families are produced from true seed families of 90–100 genotypes (Mihovilovich et al. 2017) and used to establish progeny tests in three locations, from which clonal selection can be undertaken while GCA is also estimated to advance the breeding cycles. ABS is practiced for variety development in CIP’s public–private partnership in Vietnam and was used to rapidly advance CIP’s biofortified potato population for iron and zinc concentrations (Amoros et al. 2020).

### Building sweetpotato breeding capacity

**Table:** 

Speed breeders network for sweetpotato stimulates institutional excellence	
Collaboration of CIP sweetpotato breeders in Uganda, Mozambique, Ghana and Peru with NARS via the Alliance for a Green Revolution in Africa (AGRA), and Bill & Melinda Gates Foundation SASHA I and SASHA II investments enabled East, southern and West Africa sweetpotato Support Platforms (SSPs) with focus on SPVD resistance, drought tolerance and cooking quality, respectively. The speed breeders network associated with CIP’s breeding hubs integrated germplasm collection and nutritional quality characterization with statistics, breeding methods and breeding pipelines CIP -Uganda first intensified local exploration for OFSP farmer varieties in East Africa, then established a breeding network for OFSP with five partners (Uganda, Rwanda, Kenya, Tanzania, and Mozambique) which each established polycross seed nurseries and started to incorporate local OFSP varieties into their crossing blocks, while NIRS technology was extended from CIP in Peru to Uganda for nutritional quality breeding (Grüneberg et al. 2015). A sample of about 8000 true seed of the OFSP ‘population Zapallo’ (developed by crossing advanced breeding clones from the humid tropics of Peru with South American and African varieties) was introduced to Northern Mozambique to establish a second genepool to complement previous introductions to southern Mozambique from breeding in the arid coast of Peru. These efforts in Africa resulted in the first OFSP release with moderate SPVD resistance in three East African countries SPK-004 also named Kakamega, CIP441768 (Grüneberg et al. 2015); the labeling of sweetpotato as biofortified for pro-vitamin A by HarvestPlus; and the awarding of a grant by BMGF in partnership with NARs to write a proposal for sweetpotato development in Africa for Africa (Andrade et al. 2009). Key partners were National Crops Resources Research Institute (NaCRRI; Uganda), the Mozambique Institute of Agricultural Research (IIAM), the Crops Research Institute (CSIR; Ghana) as hosts for CIP breeding platforms, and the Agricultural Research Council (ARC; South Africa), the Kenya Agricultural Research Institute (KARI), the Agricultural Research Institute (ARI; Tanzania), the Zambia Agricultural Research Institute (ZARI), the Department for Agricultural Research Services (DARS; Malawi), the Rwanda Agriculture Board (RAB), the National Root Crops Research Institute (NRCRI; Nigeria), the Ethiopian Institute of Agricultural Research (EIAR) and the Environment and Agricultural Research Institute (INERA); Burkina Faso). This effort resulted in the first Sweetpotato Action for Food Security and Health in Africa (SASHA). SASHA I aimed at developing improved breeding methods and establishing efficient population improvement programs at a sub‐regional level in SSA, linked with participatory varietal selection at the national level. Major improvements in efficiency in conventional breeding were made through the implementation of ABS, proof of exploitable heterosis (hybrid vigor), and common protocols and tools for designing and analyzing multi-locational trials. Annual speedbreeders meetings were held and all breeders trained in the use of CloneSelector software developed by CIP	

#### Heterosis exploitation

The high degree of heterozygosity in 4 × potato and 6 × sweetpotato suggests that the impact of heterosis might be very high in both crops (Bao et al. 2022). For sweetpotato, CIP breeders have demonstrated that large genetic gains can be realized by gene-pool separation and controlled recombination. We have documented very high genetic gains of from 85 to 132% for storage root yield in four OFSP H1 (hybrid 1) populations relative to the foundation after a complete reciprocal recurrent selection (RRS) cycle (Grüneberg et al. 2022). CIP presently practices a heterosis exploitation breeding scheme (HEBS), in which heterozygous parents from two mutually heterotic heterogeneous sweetpotato genepools are combined in population hybrid breeding under RRS (David et al. 2018; Diaz et al. 2021; Grüneberg et al. 2022; Swanckaert et al. 2023).

CIP’s breeding pipelines toward OFSP and WFSP with SPVD resistance in Uganda and OFSP and WFSP in Mozambique were originally improved by polycross breeding with recurrent selection (RS) using many parents (130 in Uganda and 80 in Mozambique), and long breeding cycles. In Peru, breeding for wide adapted OFSP began by RS in 2004 and started using RRS in the 2010s. All pipelines are currently utilizing controlled crossing and RRS.

CIP uses RS for potato breeding. Superior performance of inter-population hybrid families and genotypes have been found in two experimental cases, but systematic breeding scheme such as RRS to exploit this performance is not in place. A difference with sweetpotato is that, depending on cross type, progeny performance for yield is frequently greater than mid-parent value. Increased heterozygosity and more frequent multi-allelic loci contribute to increased potato yield when adapted populations or parents are crossed (Bonierbale et al. 1993; Gopal 2014; Ortiz et al. 1997). In particular, heterosis for tuber yield can result from 4*x* × 2*x* cross’s that incorporate wild or landrace germplasm into potato breeding populations by ploidy manipulation (Domański et al. 2006; Ortiz and Peloquin 1993). As for sweetpotato, the use of at least one parent with positive GCA contributes to heterosis in potato.

### True hybrid potato breeding

True hybrid potato breeding refers to the crossing of inbred parental clones to generate uniform F1 progeny as botanical seed at the diploid level (Lindhout et al. 2011). The approach takes advantage of genetic inhibition of self-incompatibility active in diploid potato, such as by the incorporation of *Sli* originally found in S. *chacoense* (Hosaka and Hanneman 1998) and recently found to be widespread in cultivated potato germplasm (Clot et al. 2020). It offers the benefits discussed earlier for TPS, with the addition of higher uniformity of true seed progenies and more straightforward introduction and dynamic stacking of new major genes due to the inbred nature of parental lines. Greatest progress has been made in the private sector and universities (Zhang et al. 2021; Jansky et al 2016; Lindhout et al. 2011; Bradshaw 2022). This breakthrough approach is accelerating scientific discovery and promises to deliver improved, true seed-propagated varieties in reduced time frames. Breeding efforts for over 10 years have advanced the status of hybrid potato, but the main breakthrough, i.e., a variety to replace any of the tetraploid varieties currently dominating the market is yet to be realized. Development of inbred lines in potato is extremely resource intensive. To reap the full benefits of this breeding method, there have been calls for potato breeding programs to form a global alliance or breeding consortium to ensure equitable sharing of genetic resources and full benefits for food security (Beumer & Stemerding 2021). These authors were concerned that hybrid potato breeding dominated by the private sector might not target traits mostly relevant for smallholder farmers, hampering the full realization of potential of hybrid breeding for food security. Establishment of a global hybrid potato breeding consortium could facilitate the development and exchange of parental lines and link actors in a successful value chain from priority setting to variety development, marketing, seed production and agronomy.

CIP has taken steps to contribute to this possible transition of potato breeding. Firstly, a new germplasm source for the induction of haploids from tetraploid potato that combines advantages of the well-known IVP stocks (Hermsen and Verdenius 1973; Hutten et al. 1994) was recently released (Ordoñez et al. 2021). Secondly, selected elite tetraploid clones were pollinated with three haploid inducers (HI) (IVP-35, IVP-101, and the above-mentioned PL-4 to initiate conversion of advanced tropically adapted populations to sets of diploid inbred parental lines. Ploidy assessment based on phenotypic markers, chloroplast counting, and flow cytometry led to the identification of dihaploid (DH) genotypes, which were assessed for agronomic attributes. Further characterization for disease resistance and tuber shape resulted in the selection of 40 DH derived from 13 diverse, elite tetraploid clones. Although the tetraploid progenitors were male fertile, the derived DH were male sterile. Initial attempts to introduce *Sli* from 97H32-6 and 97H32-14 (Phumichai et al. 2006) resulted in profusely flowering progenies with abundant and highly viable pollen, of which, however, the majority were male sterile. Our current hypothesis is that the CIP tetraploid progenitors have a fertility restorer gene that overcomes CMS, which was not inherited by the DH and that CMS affects pollen function even in the presence of *Sli*. Therefore, future attempts to develop dihaploid lines should take cytoplasmic factors into account to increase the likelihood of success. Santayana et al. (2022) recently confirmed a male fertility restoration mechanism in certain CIP breeding lines, which may prove valuable for hybrid potato breeding that makes use of CMS. Additionally, CIP has established a collaboration with private sector to enhance its capacity in deploying diploid-based breeding strategy in East-Africa.

### Molecular breeding

For both CIP crops, reference genomes have been published and high-quality assemblies are available; For potato, this was accomplished with: The Potato genome sequencing consortium 2011; Hoopes et al. 2022; Wu et al. 2018. This has greatly facilitated the identification of genomic regions of interest for the design of markers for molecular breeding. CIP breeders developed and provided the diploid progeny of ‘DM’, the doubled monoploid genotype from Virginia Tech University, which was crucial in sequencing and alignment of the first potato genomes (Sharma et al. 2013). Pan-genomes have already been developed for potato (Wang et al. 2022; Tang et al. 2022; Bozan et al. 2023) and sweetpotato assemblies have been generated for hexaploid cultivars as well as diploid relatives (reviewed by Yan et al. 2022). The phased and annotated genome of the sweetpotato variety “Beauregard” http://sweetpotato.uga.edu/, and genome sequence data of the African varieties “Tanzania”, “New Kawogo” and “Kakamega” were recently generated with the support of BMGF. In addition, reference genome sequence data of CIP varieties “Nyota” (potato) and “Benjamin” (sweetpotato) were recently generated with the support of Crops to End Hunger (CtEH) Initiative (Lindqvist-Kreuze and Mendes 2023; Lindqvist-Kreuze and Grüneberg, 2023). Thus, both crops now have the opportunity for the construction of pan-genomes, which will facilitate transfer to haplotype-based genomics assisted breeding.

CIP has also contributed to the development of molecular markers linked to key traits such as potato late blight resistance (Lindqvist-Kreuze et al. 2014, 2021), for PVY resistance (Herrera et al. 2018), and PLRV resistance (Mihovilovich et al. 2013). Such markers can have a very high return on investment particularly in the case of pathogens that are difficult to phenotype for, such as PLRV (Velásquez et al. 2007; Mihovilovich et al. 2013; Carneiro et al. 2017; RTB 2019). Further cost savings can be attributed to outsourcing of genotyping, such as has been promoted by the Excellence in Breeding Platform’s (EiB) Genotyping module (https://excellenceinbreeding.org/toolbox/collections/5). KASP (Kompetitive Allele Specific PCR, or KASP™, http://www.lgcgenomics.com) offers an excellent cost-efficient option for breeding programs to develop custom sets of low-density markers for purposes such as quality control analysis or MAS (Semagn et al. 2013; Caruana et al. 2021). Selection based on KASP markers is already mainstreamed in potato virus Y (PVY) and late blight resistance breeding and has partially replaced phenotypic screening at CIP (Kante et al. 2021). In addition, CIP has developed quality assurance markers for sweetpotato (Gemenet et al. 2020) and potato (Kante et al. 2021).

In collaboration with North Carolina State University and the University of Wisconsin, both crops are progressing with the integration of genomic selection (GS) into breeding schemes to select best parents for crosses through genomic estimated breeding values. The successful implementation of GS requires the improvement of several operational and logistical aspects and developing state-of-the-art genomic infrastructure. A cost-efficient molecular marker system has been developed for potato using DArTtag platform (https://excellenceinbreeding.org/toolbox/services/mid-density-genotyping-service) and is under development for sweetpotato. Standard operational procedures have been developed, mixed model-based statistics implemented, and database structure developed to enable the implementation of GS.

### On farm testing

Participatory research involving farmers in variety testing has traditionally been part of the CIP research for development process in both crops. The impact of participatory variety selection (PVS) in the subsequent adoption of a variety is not straightforward to estimate. However, it has been proposed that the higher-than-expected adoption rate of potato variety Canchan-INIA in Peru could be attributed at least partly to PVS (Walker 2006). Farmers were directly involved in the evaluation of candidate clones in experiments led by CIP and national partner INIA in the 1980s and were allowed to keep half of the harvest for their own purposes. After three years of testing, farmers selected the clone CIP380389.1, which was released in Peru as Canchan-INIA in 1990 (Gastelo et al. 1991). Early access to the seed had led to dozens of farmers planting the variety ahead of its release and a significant amount of seed being distributed through informal seed systems (Walker 2006). An impact study conducted after the release based on acceptance surveys predicted that the variety would reach 10 K hectares by year 2006 (Fonseca et al. 1996); however, this turned out to be an underestimate as in reality the variety reached adoption of around 58 k hectares by 2007 (Thiele et al. 2008). In the case of Canchan-INIA, the farmers also supplied the seed for the official releases as the national programs seed plot was severely damaged by frost (Walker 2006) clearly demonstrating the value of the participatory approach.

CIP has adapted and recommends participatory varietal selection using mother and baby trials that include a gender dimension for trait preferences (De Haan et al. 2019; Mudege et al. 2017). The method consists of a series of trials conducted in multiple farmers’ fields under local management practices (baby trials) and a demonstration trial that is managed centrally and with recommended agronomic practices (mother trial). Consortia of various types of institutions are united in this process, helping to articulate breeding and the selection and release and adoption of new varieties. The mother trial is evaluated by all the farmers at flowering, harvest and post-harvest and preferences are recorded based on open ended criteria as well as predetermined variables. At the end, all data are collated and utilized to select the most popular clones for variety registration trials. This approach has been frequently applied in the highland pipeline (Gastelo et al. 2021) and was recently applied to select candidate iron- and zinc-biofortified potato varieties in Peru. On farm trials were conducted in 23 locations over three years. The experience engaged 1,600 farmers and narrowed down the selection from 12 clones to the top three. The preference of farmers was taken into account, while GxE and stability analysis using linear mixed models were also employed to select the best clones.

The triadic comparison of technologies (tricot) approach (van Etten et al. 2020) has also been adopted for both CIP crops. The method consists of each farmer evaluating three anonymous varieties from a larger set of varieties tested by the community. The participants are requested to rank the three varieties as most or least preferred based on different variables such as yield and quality. The benefit of the tricot approach is the higher number of farmers that can be reached and therefore more environments tested, since not all farmers evaluate the same set of varieties (Moyo et al. 2021). Through the Bill & Melinda Gates Foundation investment SweetGAINS, tricot was mainstreamed to speed up variety registration in Uganda with promising results. The drawback of this approach is that quantitative data from the farmers are difficult to obtain.

## Dissemination strategies

### High genetic merit parents and elite true seed family distribution

Selection of progenitors on genetic merit enables distinction between the environmental and genetic components of trait expression and is thus more effective in achieving genetic gain and variety extraction than reliance on phenotypic measures only, especially when heritability is low (Dominik et al. 2017). Progeny tests from genetic mating designs have long been practiced for estimating Parental value (GCA) heritability and variance components in CIP’s breeding pipelines. This enables the identification of superior parental lines (“TPL” for tetraploid parental lines in potato; Zimnoch-Guzowska and Flis 2021), and the provision of TPL” and respective true seed families (TSF) has been a principal means of transferring CIP breeding progress to NARS. For potato, positive GCA contributed to genetic gains for tuber uniformity, crop duration, photoperiod response, PLRV resistance, late blight resistance, micronutrient density, and increased the variety ability of TSF. TPL with 3 copies of a given R gene (triplex) (Mendoza et al. 1996) for both *Ryadg* and *Rxadg* (designated TXY) have been important progenitors because they transmit complete resistance to two key viruses to virtually all of their progeny in a tetraploid breeding program.

Grüneberg et al. (2022) summarize the advantages of distributing TSF from elite crosses from clonal crop breeding programs to collaborating networks across countries as: (i) exploitation of the large segregation variance within hybrid families with true seed production on scale; (ii) rapid dissemination of genetic gains (GG) from population improvement among partners; and (iii) elimination of the time-consuming and expensive virus-cleaning procedures that are required for the international distribution of clones.

For potato, the combined evaluation of TSF and tuber families (TF) across environments allows early selection of visual, highly heritable tuber traits and testing of environmental similarities via comparative progeny performants. Feedback on performance in a short shuttle breeding project with breeders in Peru and CIP-posted agronomists on TF from TSF with different parental combinations in two environments of the subtropical Black Sea coast of Uzbekistan was applied to orient CIP’s LTVR population toward a new mid-maturity, heat and water use-efficient potato product profile when the ‘Central Asia and Caucuses’ region was established in the mid- 2000’s. Additional pressure was exerted by the determination of a tuberization index of TF through the in vitro exposure of TSF to differential temperatures and photoperiods, accompanied by parallel field evaluation of the same TSF under long days in northern China and at the threshold of daylength response for this population in southern Peru (Khan et al. 2014).

### Variety testing and dissemination

**Fig. 4:**
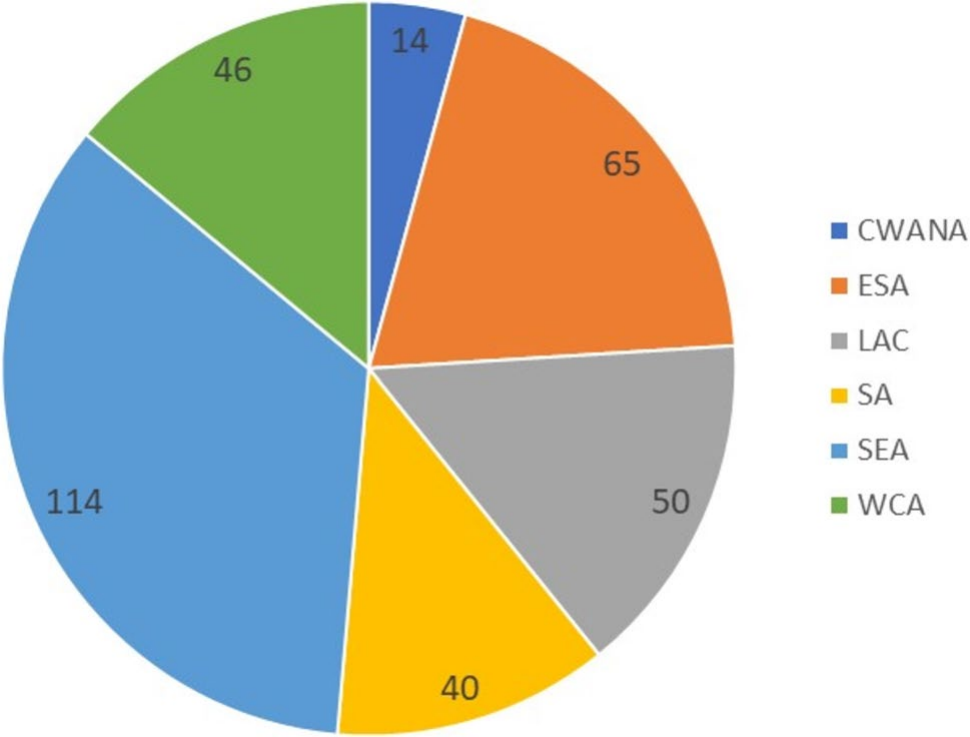
CIP-bred potato varieties (crosses made by CIP) selected and released by NARS (1982–2023) in One CGIAR regions Central West Asia and North Africa (CWANA), East and Southern Africa (ESA), Latin America and the Caribbean (LAC), South Asia (SA), South-East Asia (SEA) and West and Central Africa (WCA)

**Fig. 5:**
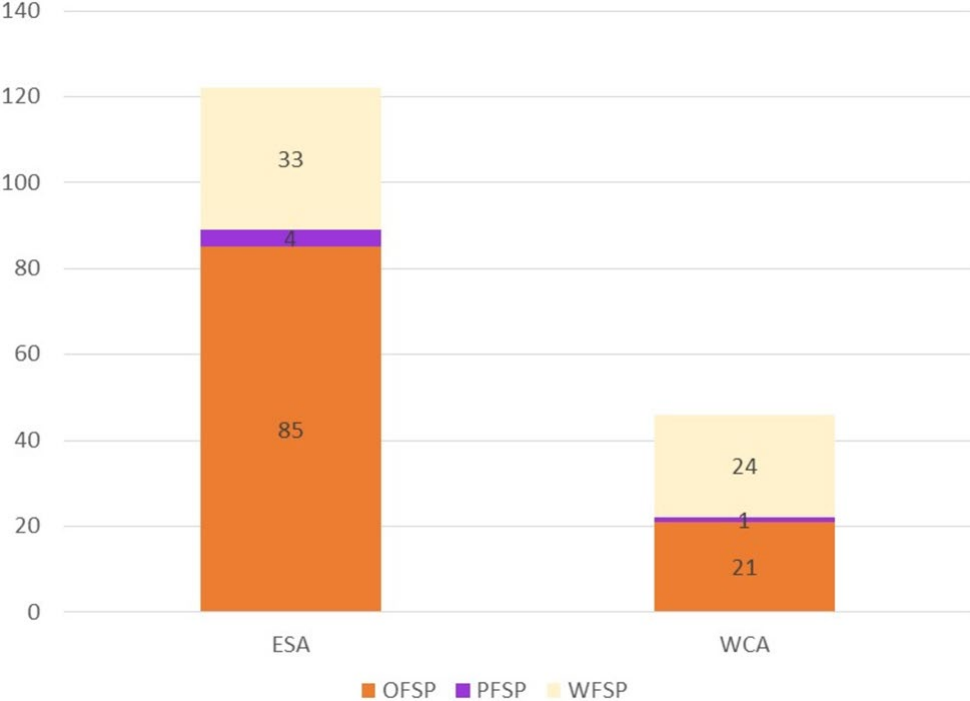
CIP bred sweetpotato varieties selected by NARS (2009–2023) in East and Southern Africa (ESA) and West and Central Africa (WCA). The varieties have the flesh colors orange (OFSP), purple (PFSP) and white (WFSP)

The distribution of potato and sweetpotato clones for variety testing is done as pathogen-free tissue culture plantlets from propagation units that have the proper authorization for distribution, such as CIP genebank in Lima or Kenya Health Inspectorate Service (KEPHIS). Thus, seed multiplication takes place under local conditions in local seed systems. Clonal crop varieties, in which there is little interest from the private sector and seed is often in short supply, face a large gap in time and capacity for seed production and dissemination following release. Dissemination to farmers can be achieved through various means, including training and awareness campaigns, seed multiplication, and establishing demonstration plots. In sweetpotato, the innovative system of “decentralized vine multipliers” incentivized the testing and farmer-to-farmer distribution of new OFSP varieties with the provision of vouchers for mother and child visits to medical clinics (Girard et al. 2021). CIP is a leading organization in potato and sweetpotato variety testing and dissemination in the tropical zones. CIP has developed, in partnership with a number of NARS, a range of improved potato (Fig. 4) and sweetpotato (Fig. 5) varieties. Some CIP-bred varieties have resulted in documented success stories in terms of impact, such as the potato clone CIP381381.20, GLIS DOI: https://doi.org/10.18730/P5MW4, having been released as a variety in Uganda, Kenya, Congo, Rwanda, Madagascar, Burundi and Malawi (Walker and Alwang 2015). In research that aimed at estimating the economic impact of CIP genebank that incorporated breeding efforts, Bernal-Galeano et al (2020) calculated the estimated gross value of this breeding line after it was released as variety ‘Victoria’ in Uganda in 1991, arriving at a benefit of USD $1.04 billion (2016 value). Potato variety “UNICA” (Gutiérrez-Rosales et al. 2016) released in Peru in 1997 has since been registered in 15 countries and has several different names, for example ‘Yusi Maap’ (Bhutan), ‘Tajikiston’ (Tajikistan), ‘Qingshu 9’ (China), ‘Kufri Uday’ (India), ‘BARI Alu-8′8 (Bangladesh), ‘Javakheturi’ (Georgia). In addition to virus resistance, UNICA has a high, stable yield in varied environments and tolerates drought. It produces quality potatoe tubers with red skin and yellow flesh that are good for fresh consumption and have the characteristics needed for French fry production. Experts estimated that more than 150,000 hectares in China were planted with ‘Qingshu 9’ in 2015 with the estimated production of 4.5 million tons. https://www.producereport.com/article/china%E2%80%99s-newest-breed-potato-produces-impressive-results; https://www.rtb.cgiar.org/news/improved-potato-variety-qingshu-9-success-story/.

By 2015, CIP had contributed to the development of 168 improved potato varieties in Asia, most of these in China (Gatto et al. 2018). This number includes varieties that NARS had selected from CIP crosses and varieties developed by NARS from CIP progenitors. Overall, in Asia, the number of direct beneficiaries of CIP-related potato varieties was estimated at 2.93 M households and indirect beneficiaries at 10.2 M households (Gatto et al. 2018). Up to July 2023, CIP records show a total of 329 potato varieties originating from CIP crosses and registered by different NARS partners globally (Fig. 4, Supplementary Table S1). It is more difficult to track varieties selected from NARS crosses with CIP progenitors and the data presented in this category in the supplementary table are likely incomplete.

According to Mwanga et al (2021), since the adoption of the accelerated breeding scheme and collaborative approach with NARS, 158 sweetpotato varieties had been released in sub-Saharan African countries. The updated number by July 2023 is a total of 168 sweetpotato varieties with purple, white and orange flesh released in 11 countries in East and Southern Africa and six countries in West and Central Africa (Fig. 5). Sweetpotato varieties released in Africa up to 2019 are also documented in the catalogue http://178.79.138.163/sweetpotato_catalogue/cip_sp_catalogue/index.php.

## Key phenomena and rationale (lessons learned)

### External review and recommendations for modernization

The most recent assessment of CIP’s breeding programs was conducted in 2018, at the headquarters in Peru and using the Breeding Program Assessment Tool (BPAT), and the areas that require strengthening were identified and an improvement plan devised. Breeding hubs in Kenya, Uganda and Mozambique were subsequently evaluated in 2021. The key components of the improvement plan included: i) re-orienting breeding activities to become more market-driven; ii) improving the definition and use of product profiles; and iii) enhancing data-driven decision processes for the advancement of new products across breeding pipelines.

Demand-driven breeding implies superimposing consumer and producer centered thinking on the agroecology-driven breeding programs and requires substantial efforts in collecting market intelligence data to understand the drivers of variety adoption. Through the 80 s, 90 s and early 2000s, CIP’s breeding priorities were developed by interdisciplinary and inter-institutional consultations led by scientists, considering constraints, opportunities and local as well as global development economics and trade. The EIB BPAT assessment recommended developing more precise market-segment oriented product profiles, explicitly and directly involving members of the value chain including NARS partners, farmers, consumers, marketers, processing companies, the seed sector and gender specialists in specifying product profiles to meet the needs and preferences of specific socio-economic market segments and value chains. This is still a work in progress and the activity is largely coordinated by two breeding-related CGIAR initiatives, namely Market Intelligence and Accelerated Breeding.

As part of an ongoing modernization effort, the breeding program is focusing on different components of the breeder’s equation (Cobb et al. 2019), such as cycle length and selection accuracy, to increase the rate of genetic gain in key traits. Selection accuracy can be improved through operational excellence, reducing errors at all levels, and increasing heritability. In addition, all CIP breeding field trials in both crops currently implement augmented p-rep designs (Williams et al. 2011) and resolvable row-column designs (Williams et al. 2006). The genotype effects are estimated through restricted maximum likelihood (REML) to allow for unbalanced data and more flexibility in the assumed variance–covariance structure. The linear mixed model approach, together with the availability of row and column plot coordinates, allow analysis that corrects for spatial variation in the field, leading to more precise estimations or predictions of the genotype treatments.

In CIP’s current strategy, improvements to data management, breeding informatics, breeding schemes, experimental designs and molecular breeding activities follow an integrated approach leveraging expertise from topic specialists. One example of this is a unified database system, wherein both crops use the Breedbase developed for tuber and root crops as a digital ecosystem for breeding data (Morales et al. 2022; Agbona et al. 2022). Android-based PhenoApps are used as compatible data collection tools to upload data to BreedBase. An example of this is FieldBook App, which has been used for recording phenotypic data in the field for the past 12 years. Digitalizing phenotypic data recording (using data dictionaries and QR coded labels to identify plots, tablets with FieldBook App for phenotypic data input, and digital Bluetooth-enabled weighing balances) has helped reduce errors and improve data accuracy. To ensure that all breeding data management procedures are standardized, standard operating procedures (SOPs) have been developed, including standardized naming conventions, data curation practices, and data workflows (International Potato Center 2020).

The present CIP breeding strategy was formulated in 2020 setting out a mission and vision for the next 10 years. To increase impact, targeting and efficiency, a fundamental change that drives our strategy since the year 2019 is the unification of previously separated crop breeding activities. Thus, we have one breeding organization delivering genetic gains potato and sweetpotato. This is welcomed by CIP breeders, since a previous review (CIP CCER–Center-commissioned Breeding Review) conducted in 1999 criticized the highlighting of opportunities for such cross-over, recommending strict adherence to separate breeding programs.

## Conclusions

### Centralized versus decentralized breeding schemes for potato and sweetpotato

Centralization of potato breeding in Peru has been effective with the most significant drawback being that many years are needed between recognition of elite clones by CIP breeders and their eventual release by NARS and use by farmers. It has been effective due to the availability of relatively uniform, well-defined worldwide quality standards, the absence of regionally critical major biotic constraints outside the center of origin where breeding has been headquartered and the proximity to several diverse and relevant environments that enabled breeding for adaptation to tropical conditions. Despite the accuracy of breeding for diverse locations in key representative sites, the bottleneck of preparing selections for international distribution, slow and sparse rates of feedback on performance, and delays in initiating seed production in target environments limit opportunities to refine hypotheses about best bet advanced materials and streamline variety testing. For example, two late blight resistant potato varieties from crosses made in 1993 were released in Bangladesh in 2014 and 2015 and have measurable adoption only now, despite having been released in other countries several years prior to their recognition in Bangladesh (Mahmud et al. 2022). Decentralization is an opportunity to increase genetic gain and investment in the regions through capacity building, and this has been successful in the case of sweetpotato. The operation of several breeding hubs that each run a full breeding program from crosses to variety development is costly but has been possible due to the large investments by BMGF for sweetpotato breeding in Africa. The Sweetpotato Action for Food Security and Health in Africa investments SASHA I in 2009 and SASHA II in 2014 were followed by the sequential BMGF investments Genomic Tools for Sweetpotato in 2015, led by NCSU, and SweetGains, led by CIP, as a follow-up phase four years later. Investment in potato breeding has come through a less regular stream of smaller, bilateral and multilateral projects, that have also supported breeding, capacity building and variety releases in at least 30 countries outside Peru.

### Pre-breeding requires constant investment and seamless integration with the breeding program

Several possible outputs of CIP’s pre-breeding research with un-adapted germplasm sources might not be brought to fruition as new varieties because only sporadic attention has been given to them, while consistent attention is needed to maintain the traits in later generations by incorporation into advance genepools and exposure to current pest or pathogen populations. If continuity is at risk or many years would be required for incorporation of pre-bred material into more usable forms, publication or germplasm releases should be undertaken to stimulate use by other programs. Expensive in vitro propagation is required for the conservation of genotypes selected from heterogeneous progeny and while breeders relied on the genebank facility to preserve selected clones from CIP and collaborating programs, this became untenable due to lack of sustained funding for the conservation of pre-bred germplasm. Changes in breeding methods, such as toward population hybrid breeding of polyploids, diploid inbred-based hybrid breeding of potato and molecular breeding promise to shorten breeding cycles and ease trait introgression and therefore should serve to increase the impact of pre-breeding.

### Toward a modern, highly effective and efficient breeding program

CIP has much benefited from regular external reviews of its breeding programs, and most recently access to evolving sets of shared services ranging from high-throughput genotyping and phenotyping to experimental design, statistical analysis and variety testing strategy as part of the EiB since its initiation in 2018. Both EiB and the Gender in Breeding initiative of the CGIAR have provided guidelines and support for exercises that are expected to improve the efficiency, effectiveness and impact of CIP’s breeding programs, such as the more accurate, interdisciplinary and cross-sector identification of market segments and design of respective breeding pipelines that efficiently address them. We believe that genomic tools, particularly GS, coupled with operational excellence will play an important role in achieving genetic gains in farmers fields. At the same time, continued investment is needed in mechanization and new phenotyping methods based on image analysis, and in innovative variety dissemination and testing schemes,

### Opportunity for greater synergies with breeding

Preparing for the future, CIP researchers have developed crop growth models, decision support systems for managing pests and diseases, and climate change (Hijmans et al. 2000; Pérez et al. 2020; Narouei-Khandan et al. 2020; Ninanya et al. 2021; Quiroz et al. 2017; Condori et al. 2010; Islam et al. 2016). They have also examined or forecasted the dynamics of pest populations (Mujica et al. 2017; Gamarra et al. 2020; Sporleder et al. 2017) and the supply and demand for food (Devaux et al. 2021;). Our prioritization and breeding methods, as well as performance forecasting for broader advantages from variety development, have been greatly influenced by this research.

However, most of our breeding has been built on its own results. The majority of our interdisciplinary collaboration has been bilateral or taking on the findings from one other discipline at a time, whereas multi-dimensional investigation and modeling would probably lead to significant advances in discovery and process development. In fact, the depth of knowledge and innovations that the integration of such information could offer are constrained by research silos and poor articulation of the enormous volumes of data gathering in various agricultural disciplines.

We anticipate that future big data integration across institutions and disciplines will enhance crops in multiple ways. For instance, techniques ranging from the integration of various genomic, GxE, and crop growth models (Messina et al. 2018; Jighly et al. 2023) to network-based machine learning (Elavarasan and Durai Raj Vincent, 2021) can likely help improve and extend predictions to direct the development and deployment of companion technologies, practices and processes that help increase productivity and resource efficiency while assisting in the adaptation of our crops to climate and demographic change.

### Sustainable and nutritious future with potato and sweetpotato

Germplasm enhancement (pre-breeding), the development of new varieties and building capacity for breeding and variety testing in changing climates with emphasis on adaptation, resistance, nutritional quality and resource-use efficiency are CIP’s central activities with significant benefits to the poor. Built on a solid foundation and embracing the latest breeding strategies, CIP’s breeding program is well positioned to continue generating better varieties for smallholders’ future needs. Both crops are already an important part of the diets of many people in the developing world, and the consumption per capita is expected to increase due to population growth. Under climate change, the crops may play even bigger role in food security because of their flexibility, resilience and short cropping cycle. Survival traits such as drought, heat and salt tolerance will be essential traits in the future and need to be combined with pest and disease resistance as well as consumer preference traits. Nutritional content, cooking time, texture and taste are increasingly important and need to be considered taking into account a gender perspective. Potato and sweetpotato breeding are important contributors to increasing productivity and nutritional quality on existing crop land. This can be achieved by integrating productive, resistant, nutritious and resource-use-efficient varieties of individual crops with interventions such as intensification and diversification. These efforts can help reduce dependence on pesticides and the frequency of crop loss while increasing systems’ productivity and decreasing the population at risk of hunger and malnutrition. Another benefit is the creation of income opportunities in marginal environments. The addition of orange-fleshed sweetpotato (OFSP) to the food environment is an effective nutrition-sensitive agricultural approach to improve vitamin A intake. These efforts can go a long way in improving the overall quality of life and health for communities that rely on these crops for their livelihoods (Low et al. 2017).

### Supplementary Information

Below is the link to the electronic supplementary material.

Supplementary file1 (XLSX 47 kb)

## Abbreviations

ARI: Advanced Research Institutes
BPAT: Breeding Program Assessment Tool
CGIAR: Consultative Group on International Agricultural Research
CIP: Centro Internacional de la papa, International Potato Center
CPRI: Central Potato Research Institute
CRP-RTB: CGIAR research program on roots, tubers and bananas
DM: Dry matter
EIB: Excellence in breeding platform
Fe: Iron
GCA: General combining ability
INIA: Institucion Nacional de Investigacion Agricola
NARS: National agricultural research system
NGO: Non-Governmental Organization
OFSP: Orange-fleshed sweetpotato
PFSP: Purple-fleshed sweetpotato
PLRV: Potato leaf roll virus
PVX: Potato virus X
PVY: Potato virus Y
SPDV: Sweetpotato virus disease
SPVD: Sweetpotato virus disease
SSA: Sub-Saharan Africa
WFSP: White-fleshed sweetpotato
